# *PROCOMIDA*, a Food-Assisted Maternal and Child Health and Nutrition Program, Contributes to Postpartum Weight Retention in Guatemala: A Cluster-Randomized Controlled Intervention Trial

**DOI:** 10.1093/jn/nxz175

**Published:** 2019-08-02

**Authors:** Jef L Leroy, Deanna K Olney, Marie T Ruel

**Affiliations:** Poverty, Health and Nutrition, International Food Policy Research Institute, Washington, DC, USA

**Keywords:** overweight, obesity, Guatemala, food aid, stunting, pregnancy, weight

## Abstract

**Background:**

Food-assisted maternal and child health and nutrition (FA-MCHN) programs are widely used to reduce household food insecurity and maternal and child undernutrition in low- and middle-income countries. These programs, however, may unintentionally lead to excessive energy intake and unhealthy weight gain, especially in food-secure populations.

**Objective:**

We evaluated the impact of an FA-MCHN program implemented in Guatemala on maternal weight from pregnancy to 24 mo postpartum. The program was earlier shown to reduce stunting.

**Methods:**

We used a longitudinal, cluster-randomized controlled trial with arms varying in family ration size [full (FFR), reduced (RFR), none (NFR)] and individual maternal ration type [corn–soy blend (CSB), lipid-based nutrient supplement (LNS), micronutrient powder (MNP)]: A: FFR + CSB; B: RFR + CSB; C: NFR + CSB; D: FFR + LNS; E: FFR + MNP; F: control. Weight was measured during pregnancy and at 1, 4, 6, 9, 12, 18, and 24 mo postpartum. We used linear mixed models controlling for pregnancy weight with random cluster and mother effects. Data on 3535 women were analyzed.

**Results:**

Significant (*P* < 0.05) or marginally significant (*P* < 0.10) effects of 0.50–0.65 kg were found at all time points (except 9 mo) in arm A. Similar-sized effects were found in arms B (1, 4, 6, and 12 mo) and C (1 and 12 mo). Marginally significant effects (0.51–0.66 kg) were found in arm D (1, 6, 9, and 12 mo); in arm E, marginally significant effects (0.48–0.75 kg) were found from 6 to 24 mo.

**Conclusions:**

The effect on maternal postpartum weight is of concern because of the high existing prevalence of overweight. Programs need to include “double-duty” objectives and actions, to ensure that addressing child undernutrition does not exacerbate the problem of unhealthy weight gain. This trial was registered at clinicaltrials.gov as NCT01072279.

## Introduction

Food-assisted maternal and child health and nutrition (FA-MCHN) programs are a widely used development strategy to reduce household hunger, food insecurity, and maternal and child undernutrition in low- and middle-income countries (1–3). These programs typically include food transfers and a package of maternal and child health and nutrition interventions including behavior change communication (BCC) and the promotion of the use of preventive health services. Recent evidence shows that these programs can effectively improve child and maternal nutrition outcomes (4–6). Food and cash transfer programs that increase household income and food access, however, have been shown to increase the risk of excessive energy intake and unhealthy weight gain, especially in energy-sufficient populations undergoing a rapid nutrition transition (7). Recent evidence from Mexico, for instance, showed that a cash and in-kind transfer program targeted to poor households living in remote rural areas significantly improved household dietary diversity, but also led to a substantial increase in household energy consumption (8). The program also increased the already steep mean annual weight gain experienced by adult women from the control group (425 g) by 68% in the group that received food transfers and by 52% in the group that received cash transfers (9).

Most countries, at all levels of development, now face a double burden of malnutrition, characterized by the coexistence of nutritional deficiencies (reflected in the presence of stunting, wasting, and micronutrient deficiencies) and overweight or obesity. This double burden of malnutrition is found at the national, community, household, and individual levels (10–13). This new nutritional reality calls for a shift from the traditional siloed approach used to address the different types of malnutrition to a more integrated strategy (7, 13, 14). Double-duty actions—defined as programs and policies that aim to simultaneously reduce the risk of both undernutrition and overweight/obesity and diet-related noncommunicable diseases—have been proposed as a holistic way to tackle malnutrition in all its forms (15). At a minimum, these double-duty actions require that current nutrition programs and policies designed to address poverty, food insecurity, and undernutrition do not inadvertently exacerbate problems of overweight and diet-related noncommunicable diseases. One of the first lines of action in the double-duty agenda is thus to document where harm is done and what could be done to prevent it from happening in the future.

The results presented here capitalize on the recently conducted evaluation of an FA-MCHN program in Guatemala. Childhood stunting in Guatemala is among the highest in the world and is found alongside a rapidly increasing prevalence of adult overweight and obesity (16, 17). The main objective of *PROCOMIDA* was to prevent undernutrition and micronutrient deficiencies in women during pregnancy and the first 6 mo postpartum, and in children aged 0–23.9 mo, through an integrated package of 3 primary components: the distribution of food rations; a BCC strategy focused on improving maternal and child health, hygiene, and nutrition practices; and improved provision and promotion of the use of health services. Program impact was evaluated using a longitudinal, cluster-randomized controlled trial with groups varying in family ration sizes—full, reduced, and none—and individual ration types provided to mothers (pregnancy to 6 mo postpartum) and children (6–24 mo of age)—corn–soy blend (CSB), lipid-based nutrient supplement (LNS), or micronutrient powder (MNP) (6). *PROCOMIDA* significantly reduced stunting at age 24 mo in the groups receiving the full family ration (FFR) combined with either CSB or MNP (by 11.1 and 6.5 percentage points, respectively). The objective of the analysis presented here was to assess whether the program had an unintended impact on maternal body weight postpartum.

## Methods

### Study population

The US Agency for International Development (USAID) Food for Peace–funded Title II FA-MCHN program (named *PROCOMIDA*) was implemented in the department of Alta Verapaz in Guatemala. Alta Verapaz is Guatemala's poorest department, with high levels of illiteracy, poor housing conditions, limited access to services like electricity, and a prevalence of stunting among children under 5 y of age of 50% (17, 18). The double burden of malnutrition is a problem in the region, with 47% of all women 15–49 y old being overweight or obese (17).

### The *PROCOMIDA* program

*PROCOMIDA* (NCT01072279) was implemented by Mercy Corps. Details on the program and the evaluation design have been published previously (6). In brief, all pregnant women in the catchment area were eligible to enroll in the program. The package of interventions included 3 core components: monthly food rations; monthly BCC sessions focused on improving health and hygiene practices, maternal nutrition, and infant and young child feeding practices; and improvements in the provision of health services and promotion of the use of these services. The program's food rations had 2 objectives: increase household food security in terms of both quantity and quality (through a family ration), and improve maternal and child nutrition (through an individual ration containing micronutrient-fortified foods or supplements targeted to pregnant women and lactating women ≤6 mo postpartum and to children starting at 6 mo of age). The program's BCC component was designed to improve health, hygiene, and nutrition practices. Participation in the monthly BCC sessions in pre- and postnatal check-ups (beneficiary mothers) and growth monitoring and promotion (beneficiary children <24 mo of age) were required to receive the monthly rations. The preventive health component consisted of training of health service providers on quality of service delivery and the promotion of use of preventive and curative health services by program participants.

### Evaluation design

*PROCOMIDA* was evaluated using a cluster-randomized controlled longitudinal study design. Cluster randomization was used because individual randomization of the treatment was not feasible. A total of 120 clusters (defined as the catchment area of a health convergence center, i.e., primary health care facilities) meeting the study criteria were randomly assigned into 1 of 6 study arms. Arm A received the FFR of rice, beans, and oil (equivalent to an estimated 269 kcal per family member per day), and CSB as the individual ration (494 kcal/d) (**Table 1**, **Supplemental Table 1**). Arm B received a reduced family ration (152 kcal per household member per day), but the same individual CSB ration (i.e., 494 kcal/d). Arm C received no family ration but the same amount of CSB as arms A and B. Arm D received the FFR and an LNS (118 kcal/d) for the individual ration. Arm E also received the FFR but MNP (no energy) for the individual ration. Arm F was the control arm, which had access to the strengthened government health services but not to the BCC sessions or food rations. All treatment arms received monthly BCC sessions delivered by trained program staff and health service–strengthening activities (**Table 2**).

**TABLE 1:** Composition of family food rations

	Full family food ration		Reduced family food ration	
	Weight (kg)	Energy (kcal)	Weight (kg)	Energy (kcal)
Rice	6.00	21,600	3.000	10,800
Beans	4.00	13,600	3.000	10,200
Vegetable oil	1.85	16,354	0.925	8177
Total	11.85	51,554	6.925	29,177
Total energy per capita, kcal/d^1^		269^2^		152^2^

1 Total energy per capita per day was calculated using a mean household size of 6.3 members (the mean household size in the enrollment survey) and 30.42 d/mo.

2 The individual ration was not meant to be shared, so it is not included in the computation of the total energy per capita per day. If the individual corn–soy blend ration was shared, it would provide an additional 78 kcal per capita per day.

**TABLE 2:** Interventions provided by *PROCOMIDA* to each treatment group^1^

	Study groups					
Program component	A (FFR + CSB)	B (RFR + CSB)	C (NFR + CSB)	D (FFR + LNS)	E (FFR + MNP)	F (Control)
Food ration						
Family ration (rice, beans, oil)	Yes	Reduced	—	Yes	Yes	—
Individual ration	Yes	Yes	Yes	Yes	Yes	—
CSB	Yes	Yes	Yes	—	—	—
LNS	—	—	—	Yes	—	—
MNP	—	—	—	—	Yes	—
BCC	Yes	Yes	Yes	Yes	Yes	—
Required health visits	Yes	Yes	Yes	Yes	Yes	—^2^

1 BCC, behavior change communication; CSB, corn–soy blend; FFR, full family ration; LNS, lipid-based nutrient supplement; MNP, micronutrient powder; NFR, no family ration; RFR, reduced family ration.

2 Households in the control group had access to the standard health services.

Women were invited to enroll in the study when they were between 3 and 7 mo pregnant; after obtaining consent from either the household head or the index mother, the enrollment interview was conducted and mothers’ weight was taken. When the child reached 1, 4, 6, 9, 12, 18, and 24 mo of age a follow-up interview was conducted and maternal weight was also measured. Study enrollment was done separately from program enrollment and generally preceded it. Inclusion in the study was based on program eligibility and not on actual program participation, allowing us to estimate the intent-to-treat effects. Study enrollment took place from August 2011 to December 2012; the last survey round (at 24 mo of age) was conducted between September 2013 and May 2015.

Sample size calculations were based on the primary study outcome, i.e., length-for-age *z* score (6). The target sample size was 600 children per study arm, with adjustments made over the enrollment period to account for attrition. Sample size at enrollment ranged from 739 to 794 pregnant women per group. We conducted a post-hoc estimation of the minimum detectable difference in body weight between arms using a type 1 error (α) of 0.05, power of 0.80, intracluster correlation coefficient of 0.01, 20 clusters per treatment arm, a CV in cluster sizes of 0.49, and an estimated explanatory power of the baseline characteristics of 0.48. The study's sample size allowed us to detect program effects of 1.25 kg. Because the explanatory power of the baseline characteristics was likely >0.48, we expect that the actual minimum detectable difference was smaller.

The protocol was approved by the Institutional Review Board of the International Food Policy Research Institute and in country by Zugueme, an independent ethics committee in Guatemala, and by Guatemala's National Ethics Committee.

### Data sources and measurement

Data were collected using computer-assisted personal interview (CAPI) software on portable computers. Surveys were programmed in Spanish and questions translated to the local language (Q'eqchi’) during each interview. Enumerators were extensively trained on interview skills, the content of the survey, and the use of the CAPI questionnaire using lectures, role-play, and discussions. Anthropometrists were trained and standardized (19). Height at study enrollment was measured twice and a third time if the difference between the first 2 measurements exceeded 10 mm. The 2 closest measurements were averaged and used in the analyses. Body weight was measured using a digital scale (SECA, model 874) and adjusted by subtracting the estimated weight of the clothes from the measured body weight. The weight of the clothes was estimated by taking the weight of a skirt and top provided by the woman and comparable in weight to the one that was currently being worn. Refresher trainings and standardization exercises were conducted periodically throughout the study.

### Statistical analysis

No clusters were dropped from the analyses. Following the CONSORT 2010 guidelines, no formal comparison of baseline means between the treatment and intervention arms was conducted (20). The key outcome variable in the analyses was women's weight. To avoid confounding due to linear growth, the program impact was estimated for women 18 y and older. Women <18 y of age (∼13% of all observations) were excluded from the analyses. Observations of women who reported becoming pregnant during the postnatal follow-up period were also excluded (i.e., observations before she reported being pregnant were included in the analyses): 3% and 12% of women reported being pregnant at 12 mo and 24 mo postpartum, respectively. We used linear mixed models with random effects (i.e., random intercepts) for health convergence center (the unit of randomization) and mother, fitting the model with restricted maximum likelihood. To assess whether the impact changed over time, a “wave × treatment” interaction term was included in the model. All models controlled for the following fixed effects: the number of household adult equivalents (the ratio of each household member's energy requirement to the energy requirement of an adult male, 18–30 y of age, was calculated and then summed), household wealth, education level of the head of household and the mother and their ability to speak Spanish, maternal age, and maternal height. The wealth index (based on ownership of household durables, housing quality characteristics, and land ownership) was created using principal components analysis (21) and quintiles of this variable were used in the analyses.

Owing to the nature of the evaluation, prepregnancy weight data could not be collected. In addition, women were enrolled in the study at different times during gestation (i.e., with different amounts of gestational weight gain) and some women (∼30%) had started receiving program benefits at the time of study enrollment. To reduce residual noise due to this heterogeneity, we controlled for weeks of gestation in our analyses and included enrollment weight as a covariate in the model. The model thus provides estimates of the program's effect net of women's weight at enrollment (i.e., during pregnancy) and thus net any pre-existing differences across study arms and the effect the program may have had by the time of study enrollment.

All observations, including those with missing data for some time points, were included in the analyses. To assess whether missing values affected the robustness of our findings, sequential multiple imputation by means of chained equations was used to fill in missing values for observations that had some information. Imputations were conducted with Stata's user-written 2-fold command, which uses Stata's “mi impute chained” command for imputation. Missing values are imputed under the missing at random assumption at a given time point using covariate information from that time point and from adjacent time points while respecting the temporal ordering of observations (22).

To confirm the results from the weight models, we estimated a mixed model with women's BMI at 12, 18, and 24 mo as the dependent variable and the same covariates as those aforementioned. All analyses were conducted using STATA 14 (StataCorp LP) and pertain to the individual level. The cut-off for statistical significance was set at a *P* value of 0.05 and for marginal significance a *P* value of 0.10 was used. All tests were 2-sided.

**FIGURE 1:**
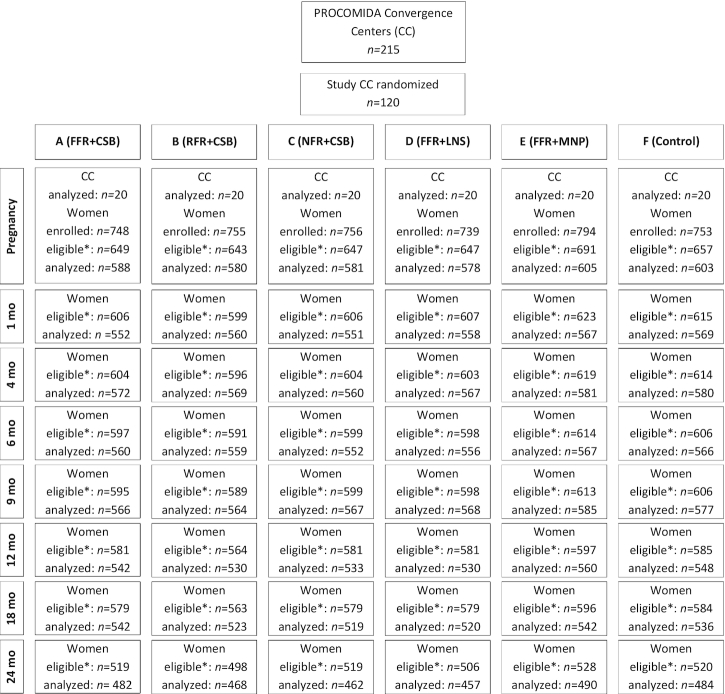
Trial profile. *Meets the eligibility criteria for the analyses presented in this article. CC, Convergence Center; CSB, corn–soy blend; FFR, full family ration; LNS, lipid-based nutrient supplement; MNP, micronutrient powder; NFR, no family ration; RFR, reduced family ration.

A total of 4545 pregnant women were enrolled in the study, 3934 of whom met the inclusion criteria for the study presented here (**Figure 1**). Of these pregnant women, 3535 (or 90%) had complete data and were included in the analyses. For all follow-up time points, the percentage of observations excluded because of missing values was <10%. By 24 mo postpartum, 3090 women were still eligible for inclusion and 2843 (or 92%) of those had complete data and were actually included in the analyses.

## Results

Households had a mean of 6 members (**Table 3**). Household hunger was uncommon, affecting <10% of the study population. Living conditions were suboptimal. The majority of households lived in houses with dirt floors and only 1 in 4 households had electricity. Only half of the heads of household and ∼40% of pregnant women had attended school. Baseline characteristics were balanced across trial arms.

**TABLE 3:** Unadjusted mean characteristics of households and mothers included in the study sample by treatment group^1^

	Study arms					
	A (FFR + CSB)	B (RFR + CSB)	C (NFR + CSB)	D (FFR + LNS)	E (FFR + MNP)	F (Control)
*n*	588	580	581	578	605	603
Household (at enrollment)						
Size	6.1 ± 3.0	6.0 ± 2.9	6.1 ± 2.9	6.4 ± 2.9	5.8 ± 2.8	6.0 ± 2.9
Dirt floor	89.3	84.0	79.7	80.6	81.3	86.7
Wood walls	66.8	68.5	64.2	68.7	76.7	75.0
Electricity	20.8	26.2	27.4	28.6	25.6	15.8
Moderate or severe hunger	7.5	10.7	9.5	5.0	5.5	9.0
Head has no education	49.5	45.9	43.4	36.7	46.6	43.1
Head speaks Spanish	42.5	42.8	44.4	52.1	46.1	43.0
Mother						
Age (at enrollment), y	26.0 ± 6.1	26.1 ± 5.7	25.9 ± 5.8	26.2 ± 5.8	26.2 ± 6.0	26.3 ± 6.1
No education (at enrollment)	40.3	41.0	37.4	31.8	39.7	36.0
Speaks Spanish (at enrollment)	25.2	31.0	28.6	38.8	27.3	26.7
Weeks pregnant (at enrollment)	22.9 ± 5.5	22.5 ± 5.6	23.1 ± 5.9	23.5 ± 5.8	22.8 ± 5.6	22.0 ± 5.9
BMI (at 24 mo)	24.4 ± 3.3	24.2 ± 3.3	24.1 ± 3.3	24.4 ± 3.7	23.8 ± 3.2	23.8 ± 3.3
Underweight (at 24 mo)	0.6	0.9	0.7	0.4	1.0	1.7
Overweight (at 24 mo)	28.2	28.2	23.8	28.9	22.2	24.4
Obese (at 24 mo)	6.4	5.6	5.4	7.9	5.5	5.2
Time postpartum, mo						
1-mo wave	1.2 ± 0.4	1.2 ± 0.5	1.2 ± 0.5	1.3 ± 0.6	1.2 ± 0.5	1.2 ± 0.4
4-mo wave	4.1 ± 0.3	4.1 ± 0.2	4.1 ± 0.3	4.1 ± 0.3	4.1 ± 0.2	4.1 ± 0.3
6-mo wave	6.1 ± 0.3	6.1 ± 0.2	6.1 ± 0.2	6.1 ± 0.2	6.1 ± 0.2	6.1 ± 0.3
9-mo wave	9.1 ± 0.2	9.1 ± 0.2	9.1 ± 0.2	9.1 ± 0.3	9.1 ± 0.2	9.1 ± 0.2
12-mo wave	12.0 ± 0.2	12.0 ± 0.3	12.0 ± 0.2	12.1 ± 0.4	12.0 ± 0.2	12.0 ± 0.1
18-mo wave	18.0 ± 0.2	18.0 ± 0.1	18.0 ± 0.1	18.0 ± 0.1	18.0 ± 0.1	18.0 ± 0.1
24-mo wave	24.0 ± 0.2	24.0 ± 0.2	24.0 ± 0.2	24.0 ± 0.2	24.0 ± 0.2	24.0 ± 0.1
Enrolled in *PROCOMIDA*						
Study enrollment	36.4	32.8	28.8	37.8	29.1	0.3
1-mo wave	69.8	68.4	54.8	69.9	62.6	4.4
4-mo wave	81.8	78.4	59.5	79.5	74.9	1.6
6-mo wave	85.3	80.8	62.6	84.8	79.8	2.8
9-mo wave	86.6	83.7	64.7	85.8	81.4	2.9
12-mo wave	86.5	84.4	63.6	85.6	82.4	2.9
18-mo wave	85.3	85.2	59.3	85.5	82.7	2.2
24-mo wave	82.8	82.1	50.4	84.7	79.4	2.5
Among beneficiaries, number of food rations received…^2^						
Since birth (at 4-mo survey)	3.3 ± 1.0	3.4 ± 0.9	3.0 ± 0.9	3.2 ± 0.9	3.2 ± 1.9	3.4 ± 1.1
Since 4 mo (at 6-mo survey)	1.9 ± 0.4	1.9 ± 0.5	1.8 ± 0.6	1.9 ± 0.4	1.9 ± 0.5	1.8 ± 0.5
Since 6 mo (at 9-mo survey)	2.9 ± 0.5	2.8 ± 0.5	2.6 ± 0.7	2.9 ± 0.4	2.8 ± 0.5	2.8 ± 0.6
Since 9 mo (at 12-mo survey)	2.9 ± 0.4	2.8 ± 0.6	2.5 ± 0.7	2.8 ± 0.5	2.8 ± 0.6	2.6 ± 1.0
Since 12 mo (at 18-mo survey)	5.7 ± 0.9	5.7 ± 0.9	5.2 ± 1.3	5.6 ± 1.0	5.7 ± 0.9	5.3 ± 1.4
Since 18 mo (at 24-mo survey)	5.8 ± 0.8	5.7 ± 0.8	5.3 ± 1.2	5.8 ± 0.7	5.7 ± 0.8	5.8 ± 0.9

1 Values are means ± SDs or percentages unless otherwise indicated. CSB, corn–soy blend; FFR, full family ration; LNS, lipid-based nutrient supplement; MNP, micronutrient powder; NFR, no family ration; RFR, reduced family ration.

2 The expected number of rations received equals the difference between the survey rounds. At the 6-mo survey, for instance, households could have received 2 rations since the 4-mo survey.

At study enrollment, women were ∼5 mo pregnant and 1 out of 3 was enrolled in the *PROCOMIDA* program. Program participation increased to >80% by month 4 postpartum, except in arm C (which did not receive a family ration), where enrollment stayed <65%. Beneficiary households generally reported receiving food rations according to schedule, i.e., once a month.

**FIGURE 2:**
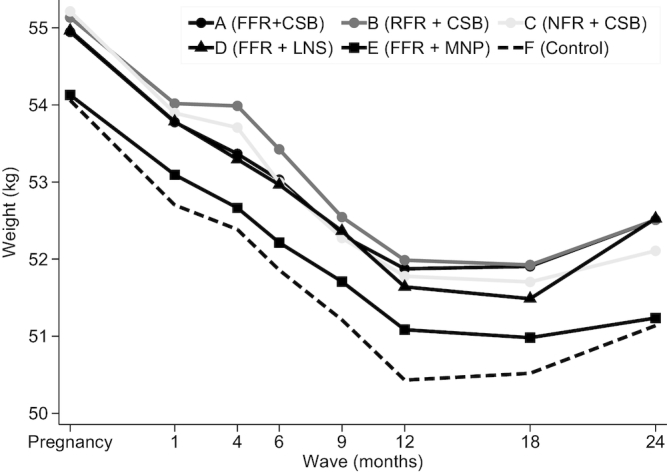
Mean unadjusted weight by wave and treatment arm of women from Alta Verapaz, Guatemala enrolled in the evaluation study of the *PROCOMIDA* program. Weight data from a total of 26,376 observations were included. Solid lines are intervention arms and the dashed line is the control arm. The darkness of the line and marker reflects the size of the family ration (darker shades indicating a larger ration) and the marker type shows the type of individual ration (circle: CSB; triangle: LNS; square: MNP). CSB, corn–soy blend; FFR, full family ration; LNS, lipid-based nutrient supplement; MNP, micronutrient powder; NFR, no family ration; RFR, reduced family ration.

**FIGURE 3:**
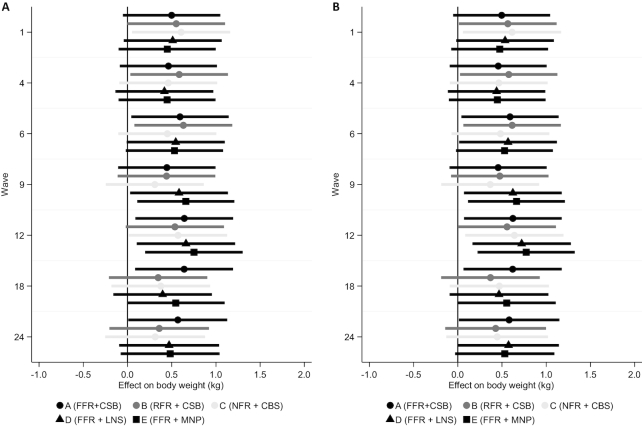
Impact of *PROCOMIDA* in Alta Verapaz, Guatemala on women's weight using linear mixed models with random effects for health convergence center (the unit of randomization) and mother. Values shown are wave- and arm-specific impact estimates and 95% CIs. (A) Impact estimates using all nonmissing observations (a total of 22,555 observations). (B) Impact estimates using imputed data (a total of 24,014 observations). The darkness of the line and marker reflects the size of the family ration (darker shades indicating a larger ration) and the marker type shows the type of individual ration (circle: CSB; triangle: LNS; square: MNP). CSB, corn–soy blend; FFR, full family ration; LNS, lipid-based nutrient supplement; MNP, micronutrient powder; NFR, no family ration; RFR, reduced family ration.

Women's unadjusted weight declined from 1 mo up to 12 mo, after which it reached a plateau and started to increase at 18 mo postpartum (**Figure 2**). *PROCOMIDA* had a significant impact on women's body weight (**Figure 3**A, **Supplemental Table 2**). Program impacts of 0.50–0.65 kg were found to be significant or marginally significant in the A arm, except at 9 mo. Similar effects (ranging from 0.55 to 0.63 kg) were found in the B arm at 1, 4, 6, and 12 mo. In the C arm, *PROCOMIDA*’s impact was limited to 1 (0.61 kg) and 12 mo (0.57 kg). The program had a significant or marginally significant impact on women's postpartum weight of 0.51–0.66 kg at 1, 6, 9, and 12 mo in the D arm. In the E arm (marginally) significant effects ranging in size from 0.48 to 0.75 kg were found from 6 to 24 mo. The impact estimates using imputed values where data were missing (Figure 3B, Supplemental Table 2) were on average slightly larger and more of the estimates were statistically significant.

The results of the BMI model show that at 12 mo postpartum, the program had a significant impact on women's BMI (in kg/m^2^) of 0.34, 0.32, and 0.41 in arms A, D, and E, respectively (**Supplemental Figure 1**). At 18 mo, the impact was marginally significant in arm A only and at 24 mo no significant effect was found.

## Discussion

Using a rigorous study design, our study demonstrates that an FA-MCHN program, aiming to reduce childhood undernutrition and implemented in an area with a high prevalence of overweight and obesity, increased women's weight in the perinatal period. In the standard program, i.e., the arm receiving the FFR and CSB as the individual ration, the program had an overall estimated effect of nearly 600 g at 24 mo postpartum. The results suggest a positive relation between the size of the family ration and the impact on women's weight. The largest program impact and the largest number of significant effects were found in the standard program. Smaller effects on weight, and at fewer time points, were found with the smaller family rations.

The program's impact on body weight is of concern because of the pre-existing problem of overweight and obesity in this population. A cross-sectional survey we conducted in the study area in May 2010 (∼1 y before recruitment started for the study reported here) found that 42.5% of nonpregnant women were overweight or obese (18). Because unhealthy weight is associated with higher all-cause mortality (29) and other negative health outcomes, the higher mean weight found in the intervention compared with the control arm suggests that the program unintentionally caused harm for beneficiary women. An additional concern is the large size of the effect. Using women's mean height, the weight impacts are equivalent to an increase in mean BMI of 0.26–0.27, similar to the BMI impact estimate of 0.24. Mean BMI in Guatemalan women has increased nationally from 22.9 in 1980 to 26.8 in 2008 (16), or by 0.14/y. This is equivalent to an expected change of 0.35 over ∼30 mo of program exposure (6 mo of pregnancy and 24 mo of postpartum). The *PROCOMIDA* program thus roughly doubled the already steep secular trend in weight gain.

What are the possible underlying mechanisms explaining this impact on women's weight? It is unlikely that the program's impact on maternal weight was a consequence of a decrease in physical activity levels. An alternative potential explanation is that the rations reduced breastfeeding and breastfeeding-induced postpartum weight loss. *PROCOMIDA*, however, significantly improved breastfeeding practices, making this an improbable pathway of impact.

The association between the size of the family ration and the size of the impact on weight suggests that the effect was a consequence of an increase in women's dietary energy intake. A qualitative substudy nested in the evaluation trial showed that, compared with the control group, program beneficiary families consumed program ration foods (rice, red beans, and oil) more frequently; beneficiaries reported consuming eggs, local plants, and some vegetables more frequently, but also increased their consumption of energy-dense foods such as pasta and sugar (23). Current knowledge on the mechanisms that help explain the behavior of increasing energy intake in energy-sufficient households is limited. Economic theory of household decision making predicts that households receiving food rations will reduce purchases of the foods in the ration and will subsequently use the freed-up resources to increase consumption of other higher-quality foods or of nonfoods (24). Empirical evidence shows, however, that these “substitution” effects are not smooth. Food transfers tend to lead to “stickiness” in food consumption, resulting in a larger increase in consumption of the transferred foods than predicted by theory. This may cause transfers of staple food rations to increase calorie intake. Key reasons for this behavior identified in the literature include transaction costs (the cost of selling the received food) and “labeling” effects (households inferring that more of the transferred foods should be consumed) (25, 26).

The impact estimates at different time points provide useful insights into the time dynamics of the program's impact. Our analysis of the change in impact with time postpartum suggests that the largest weight effect happened early on: significant effects were evident at 1 mo postpartum and grew by no more than an additional 30% by the time women were 12 mo postpartum. By 24 mo, a significant (or marginally significant) program impact was only found in the arms receiving the FFR. Statistical power, however, may have limited our ability to detect a program impact in the other arms.

Our study cannot assess whether or how fast women returned to their prepregnancy weight. Interestingly, there is no current official guidance on how quickly women should return to this weight. A study in Danish women showed that women with a reasonable amount of weight gain (∼12 kg), who exclusively breastfed their infants for the first 6 mo, returned to their prepregnant weight by 6 mo postpartum (27). The rate at which a woman returned to her prepregnant weight was found to depend on 3 factors: her BMI before conception, the amount of weight she gained during pregnancy, and the intensity and duration of breastfeeding (27). No data are available on the first 2 factors in this study. The cross-sectional survey we conducted in the study area shortly before the start of the longitudinal survey, however, found that 42.5% of nonpregnant women were overweight or obese. We can thus assume that a large proportion of women in the longitudinal study had an unhealthy weight before conception. Using the variation in gestational age at enrollment provides information on the second factor, i.e., gestational weight gain. We found that estimated weekly weight gain was only ∼233 g between 15 and 30 wk, which is well below the Institute of Medicine–recommended 400 g/wk and 300 g/wk in both the second and third trimesters for women with normal and high prepregnancy BMI, respectively (28). Other research in rural Guatemalan women with a high prevalence of overweight and obesity has similarly documented low gestational weight gain (29). The third factor, exclusive breastfeeding, was high in our study population: 94% of mothers reported exclusively breastfeeding their child at 1 mo and 86% at 4 mo; over 95% of mothers were still breastfeeding their child at 12 mo. The high prevalence of overweight, the below-normal gestational weight gain, and the already high breastfeeding rates in this population make formulating recommendations for healthy gestational weight gain and postnatal weight loss challenging.

A limitation of our analytical approach is that by including enrollment weight as a covariate, we may have underestimated the impact of the program on women's weight. Because only ∼30% of women were program beneficiaries at study enrollment, we believe that the size of the potential bias is small. When we estimated the impact model without the women who were program beneficiaries at study enrollment, the impact point estimates were somewhat smaller at 1 mo in the 3 arms receiving CSB (arms A, B, and C) and in the LNS arm (D), but not in the MNP arm (E) (**Supplemental Table 3**). This could suggest that we underestimated early program impact in these 4 arms. No other systematic differences were found later postpartum. The validity of this model is limited, however, because the estimates are based on a nonrandomly selected group in the treatment arms (i.e., those who were *PROCOMIDA* beneficiaries at study enrollment), without being able to do the same in the control group (i.e., selecting women who would have been in the program if it had been available to them).

Our findings have important implications for programs. The intervention package that resulted in the highest program participation and was most successful and cost-effective at reducing stunting was also the most damaging with respect to maternal weight at 24 mo postpartum (6). The intervention area was selected because of its high stunting prevalence. Our results demonstrate that in contexts like rural Guatemala where the nutrition transition is accelerating, program planners should carefully consider the trade-offs related to the types and quantities of food provided to maximize program participation and impact on child nutritional status without exacerbating the problem of maternal overweight and obesity. FA-MCHN programs implemented in these areas will thus have to widen their scope to include “double-duty” objectives and actions. The BCC component of FA-MCHN programs should promote the consumption of balanced and diverse diets composed of micronutrient-rich foods as a determinant of healthy pregnancy weight gain and postpartum weight loss. They should also explore whether other transfer approaches (such as cash or vouchers that can be used for fresh food purchases) combined with strong BCC lead to more desirable outcomes. Finally, FA-MCHN evaluation studies should be powered to detect meaningful impacts on weight gain.

## Supplementary Material

nxz175_Supplemental_File

## Notes

The study was made possible by the generous support of the American people through the support of the Office of Food for Peace, Bureau for Democracy, Conflict, and Humanitarian Assistance, and the Office of Health, Infectious Diseases, and Nutrition, Bureau for Global Health, US Agency for International Development (USAID), under terms of Cooperative Agreement No. AID-OAA-A-12-00005, through the Food and Nutrition Technical Assistance III Project (FANTA), managed by FHI 360. This study also received support from the CGIAR Research Program on Agriculture for Nutrition and Health (A4NH), led by the International Food Policy Research Institute.

Author disclosures: JLL, DKO, and MTR, no conflicts of interest.

Supplemental Tables 1–3 and Supplemental Figure 1 are available from the “Supplementary data” link in the online posting of the article and from the same link in the online table of contents at https://academic.oup.com/jn/.

Abbreviations used: BCC, behavior change communication; CAPI, computer-assisted personal interview; CSB, corn–soy blend; FA-MCHN, food-assisted maternal and child health and nutrition; FFR, full family ration; LNS, lipid-based nutrient supplement; MNP, micronutrient powder; NFR, no family ration; RFR, reduced family ration; USAID, United States Agency for International Development.
