# Supplementary material for: PROCOMIDA, a Food-Assisted Maternal and Child Health and Nutrition Program, Contributes to Postpartum Weight Retention in Guatemala: A Cluster-Randomized Controlled Intervention Trial
Source: J Nutr. 2019 Aug 2;149(12):2219–27. doi: 10.1093/jn/nxz175 (PMC6888017; doi:10.1093/jn/nxz175)

Supplemental Table 1 Composition of daily dose of CSB, lipid-based nutrient supplement (LNS), and micronutrient powder (MNP)

	Unit	CSB	LNS		MNP	
			Child	Mother	Child	Mother
Daily dose		132 g[1]	20 g (two 10 g sachets)	20 g (1 sachet)	4 g (two 2 g sachets)	4 g (two 2 g sachets)
Energy	kcal	494	118	118	–	–
Proteins	g	22.6	2.6	2.6	–	–
Fat	g	9.1	9.6	10.0	–	–
Linoleic acid	g	–	4.46	4.60	–	–
α-Linolenic acid	g	–	0.58	0.60	–	–
Vitamin A	µg	1031	400	800	400	800
Vitamin C	mg	53	30	100	30	100
Vitamin D	µg	6.5	5	10	5	10
Vitamin E	mg	11.4	6	20	6	20
Vitamin K	µg	–	30	45	30	45
Thiamine (B1)	mg	0.7	0.5	2.8	0.5	2.8
Riboflavin (B2)	mg	0.6	0.5	2.8	0.5	2.8
Niacin	mg	8	6	36	6	36
Pantothenic acid (B5)	mg	4.5	2	7	2	7
Vitamin B6	mg	0.7	0.5	3.8	0.5	3.8
Folic Acid	µg	395	150	400	150	400
Vitamin B12	µg	1.3	0.9	5.2	0.9	5.2
Iron	mg	23	9	20	9	20
Zinc	mg	7	8	30	8	30
Copper	mg	1.20	0.34	4.00	0.34	4.00
Selenium	µg	8	20	130	20	130
Iodine	µg	75	90	250	90	250
Calcium	mg	1093	280	280	280	280
Magnesium	mg	229	40	65	40	65
Manganese	mg	0.9	1.2	2.6	1.2	2.6
Phosphorus	mg	271	190	190	190	190
Potassium	mg	834	200	200	200	200

[1] Beneficiaries received 4 kg of CSB per month or an average of 132 g per day.

Supplemental Table 2 Impact of PROCOMIDA on women's weight (kg) using linear mixed models with random effects for health convergence center (the unit of randomization). Impact was estimated relative to the control group, using all non-missing observations (model 1) and using imputed data (Model 2). The impact estimates are shown in Figure 3 in the text.

	Model 1[1, 2]					Model 2[1,2]				
	Treatment arm					Treatment arm				
	A (FFR + CSB)	B (RFR + CSB)	C (NFR + CSB)	D (FFR + LNS)	E (FFR + MNP)	A (FFR + CSB)	B (RFR + CSB)	C (NFR + CSB)	D (FFR + LNS)	E (FFR + MNP)
1 month	0.498*	0.550*	0.606**	0.511*	0.449	0.496*	0.563**	0.611**	0.534*	0.474*
	(0.281)	(0.282)	(0.283)	(0.283)	(0.280)	(0.281)	(0.282)	(0.283)	(0.282)	(0.280)
4 months	0.462*	0.584**	0.460	0.417	0.447	0.452	0.573**	0.464*	0.431	0.441
	(0.280)	(0.282)	(0.282)	(0.282)	(0.280)	(0.279)	(0.280)	(0.282)	(0.281)	(0.279)
6 months	0.593**	0.631**	0.450	0.545*	0.530*	0.586**	0.614**	0.486*	0.567**	0.528*
	(0.281)	(0.282)	(0.283)	(0.283)	(0.281)	(0.280)	(0.281)	(0.282)	(0.282)	(0.280)
9 months	0.445	0.441	0.308	0.583**	0.659**	0.448	0.474*	0.367	0.618**	0.662**
	(0.281)	(0.282)	(0.283)	(0.283)	(0.280)	(0.280)	(0.281)	(0.282)	(0.282)	(0.280)
12 months	0.642**	0.536*	0.570**	0.661**	0.751***	0.622**	0.570**	0.645**	0.724**	0.776**
	(0.282)	(0.283)	(0.284)	(0.284)	(0.281)	(0.282)	(0.283)	(0.284)	(0.283)	(0.281)
18 months	0.640**	0.346	0.376	0.397	0.545*	0.624**	0.381	0.485*	0.475*	0.556**
	(0.282)	(0.284)	(0.285)	(0.285)	(0.282)	(0.282)	(0.284)	(0.285)	(0.285)	(0.282)
24 months	0.568**	0.358	0.312	0.470	0.482*	0.583**	0.423	0.441	0.581**	0.527*
	(0.286)	(0.287)	(0.288)	(0.288)	(0.285)	(0.290)	(0.290)	(0.293)	(0.292)	(0.288)

[1] Values shown are wave and arm-specific impact estimates and standard errors.

[2] A total of 22,555 and 24,014 observations were included in model 1 and 2, respectively.

*** p<0.01, ** p<0.05, * p<0.1

Supplemental Table 3 Impact of PROCOMIDA on women's weight (kg) using linear mixed models with random effects for health convergence center (the unit of randomization) and mother using non-missing observations but excluding mothers who were enrolled in the program at baseline.

	Treatment arm[1,2]				
	**A (FFR + CSB)**	**B (RFR + CSB)**	**C (NFR + CSB)**	**D (FFR + LNS)**	**E (FFR + MNP)**
1 month	0.374	0.349	0.526*	0.334	0.444
	(0.303)	(0.300)	(0.299)	(0.304)	(0.296)
4 months	0.459	0.443	0.450	0.299	0.420
	(0.303)	(0.300)	(0.298)	(0.304)	(0.295)
6 months	0.597**	0.561*	0.486	0.491	0.641**
	(0.304)	(0.300)	(0.299)	(0.305)	(0.296)
9 months	0.426	0.353	0.249	0.545*	0.718**
	(0.303)	(0.300)	(0.299)	(0.304)	(0.295)
12 months	0.496	0.393	0.486	0.578*	0.828***
	(0.305)	(0.302)	(0.300)	(0.307)	(0.297)
18 months	0.607**	0.256	0.366	0.335	0.634**
	(0.305)	(0.302)	(0.301)	(0.307)	(0.298)
24 months	0.536*	0.220	0.351	0.482	0.554*
	(0.309)	(0.306)	(0.306)	(0.312)	(0.301)

[1] Values shown are wave and arm-specific impact estimates and standard errors.

[2] A total of 16,396 observations were included in model 1 and 2, respectively.

*** p<0.01, ** p<0.05, * p<0.1

Supplemental Figure 1: Impact of PROCOMIDA on women's BMI at 12, 18, and 24 mo postpartum using linear mixed models with random effects for health convergence center (the unit of randomization) and mother. A total of 9,216 observations were included in the analysis. Values shown are wave- and arm-specific impact estimates and 95% confidence intervals. The darkness of the line and marker reflects the size of the family ration (darker shades indicating a larger ration) and the marker type shows the type of individual ration (circle: CSB; triangle: LNS; square: MNP).

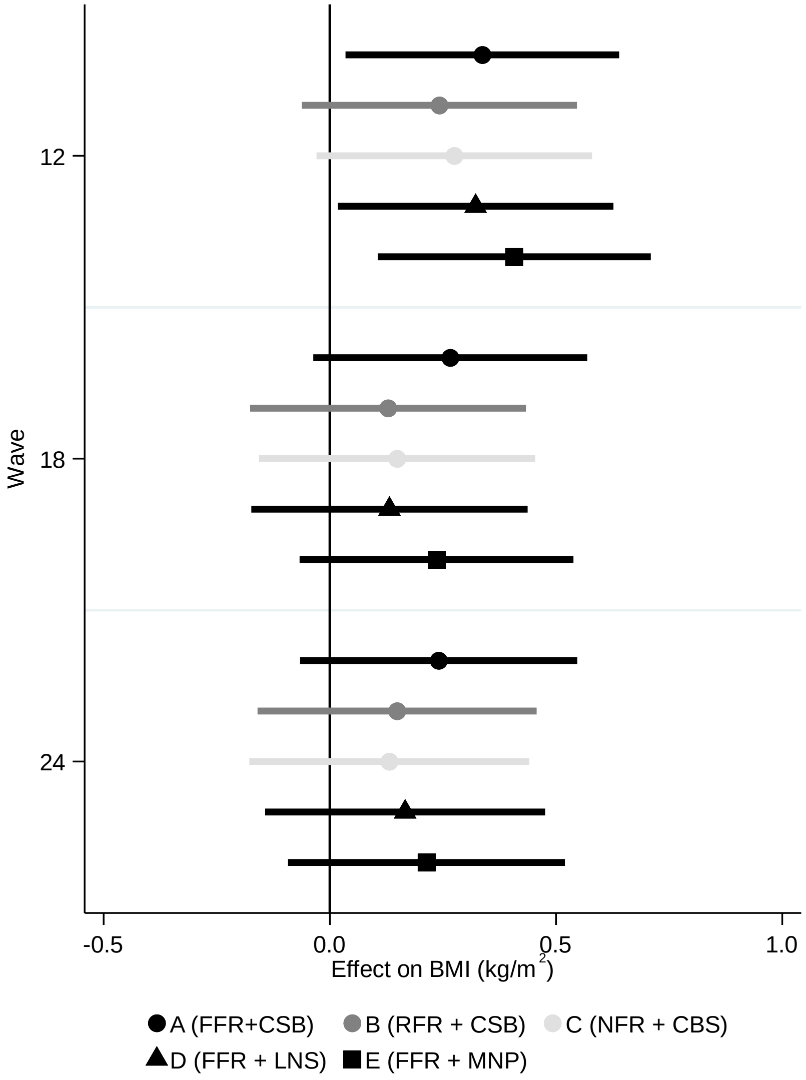

Supplement: nxz175_Supplemental_File [file nxz175_supplemental_file.pdf]
